# An mRNA decapping mutant deficient in P body assembly limits mRNA stabilization in response to osmotic stress

**DOI:** 10.1038/srep44395

**Published:** 2017-03-14

**Authors:** Susanne Huch, Tracy Nissan

**Affiliations:** 1 Department of Molecular Biology, Umeå University, SE-901 87, Umeå, Sweden

## Abstract

Yeast is exposed to changing environmental conditions and must adapt its genetic program to provide a homeostatic intracellular environment. An important stress for yeast in the wild is high osmolarity. A key response to this stress is increased mRNA stability primarily by the inhibition of deadenylation. We previously demonstrated that mutations in decapping activators (*edc3∆ lsm4∆C*), which result in defects in P body assembly, can destabilize mRNA under unstressed conditions. We wished to examine whether mRNA would be destabilized in the *edc3∆ lsm4∆C* mutant as compared to the wild-type in response to osmotic stress, when P bodies are intense and numerous. Our results show that the *edc3∆ lsm4∆C* mutant limits the mRNA stability in response to osmotic stress, while the magnitude of stabilization was similar as compared to the wild-type. The reduced mRNA stability in the *edc3∆ lsm4∆C* mutant was correlated with a shorter *PGK1* poly(A) tail. Similarly, the *MFA2* mRNA was more rapidly deadenylated as well as significantly stabilized in the *ccr4∆* deadenylation mutant in the *edc3∆ lsm4∆C* background. These results suggest a role for these decapping factors in stabilizing mRNA and may implicate P bodies as sites of reduced mRNA degradation.

A change in environmental osmolarity is a fundamental stress for cells as they strive to maintain consistent intracellular osmolarity to allow proper cellular functioning. Single-celled organisms that possess motility can avoid unfavourable osmotic conditions by altering their location, whereas multicellular organisms often maintain constant osmolarity in their bodily fluids. For example, osmolarity is maintained at a constant range in humans by the kidneys, maintaining a normal variation of less than three per cent^1^. In contrast to these organisms, the free-living yeast *Saccharomyces cerevisiae* must adapt rapidly to environmental changes to maintain intracellular osmotic homeostasis. An example of these rapid changes in extracellular osmolarity includes the transition from an isotonic to a hypo-osmotic environment by being washed off a fruit in the rain. Alternatively, yeast can be exposed to high osmolarity environments arising from, for example, the high sugar content of ruptured fruit. These types of changes require rapid sensing and adaptation to maintain intracellular osmotic homeostasis^2^.

Yeast has several key responses to changes in osmolarity. An initial response to a hyper-osmotic shock results in the inhibition of translation within 10–15 minutes, which can persist for an hour or more depending on its intensity^3^,^4^,^5^,^6^. Similarly, the abundance of specific mRNAs shows the greatest change at a similar time-scale^7^,^8^,^9^,^10^. Up until the last ten years, gene expression changes were largely thought to be due to transcriptional changes; since then the significance of mRNA stability in response to osmotic stress has been more appreciated from experiments performed in yeast as well as in human cell lines^11^,^12^,^13^,^14^,^15^. The modulation of mRNA degradation can vary between weak and strong osmotic stresses. Weaker stress tends to destabilize mRNAs, whereas stronger stress results in mRNA stabilization^11^,^12^,^13^,^14^. The stabilization occurs primarily as a result of inhibition of deadenylation^11^,^13^,^14^. Concomitant with these changes are the formation of P bodies, which are cytoplasmic RNA granule foci composed of non-translating mRNA, mRNA decay intermediates and mRNA degradation factors^14^,^16^.

P bodies contain factors involved in deadenylation, the initial stage of mRNA degradation, as well as the components of the decapping-dependent 5′-to-3′ mRNA decay pathway^16^,^17^,^18^,^19^,^20^. A yeast mutant has been identified that functions in P body assembly^19^. It lacks the decapping activating protein Edc3 as well as the glutamine/asparagine rich C-terminal tail of Lsm4, which is a component of both the Lsm1-7 decapping activator complex and the Lsm2-8 complex involved in splicing. We have previously demonstrated that this mutant (*edc3∆ lsm4∆C*) reduces mRNA stability without additional stress^21^. Because this mutant has been credited with a role in P body formation and has an effect on mRNA stability, we wished to examine whether the *edc3∆ lsm4∆C* mutant would contribute to mRNA stability in response to osmotic stress. Our results demonstrate that the *edc3∆ lsm4∆C* mutant has reduced mRNA stabilization in response to osmotic stress for all of the mRNAs we examined. However, the fold stabilization upon hyper-osmotic stress was similar in both the wild-type and *edc3∆ lsm4∆C* mutant. By examining mRNA degradation mutants and poly(A) length, these results suggest that mRNA deadenylation can be affected in the *edc3∆ lsm4∆C* mutant, which results in more rapid mRNA degradation. Finally, although we did not observe a significant role for the 3′-to-5′ mRNA degradation pathway in the *edc3∆ lsm4∆C* mutant in response to osmotic stress our results suggest that it could play an important role in the degradation of mRNA found in P bodies.

## Results

### The *edc3∆ lsm4∆C* mutant is deficient in P body formation under glucose starvation or osmotic stress

**Figure 1:**
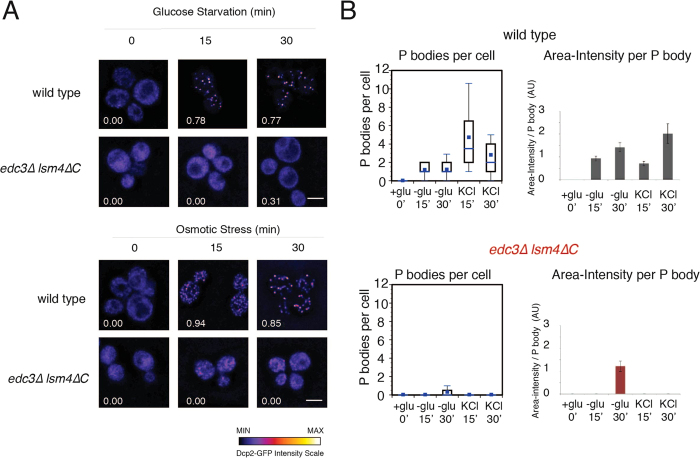
P body characterization in the wild-type and *edc3∆ lsm4∆C* mutant strains deficient in P body formation. (**A**) Yeast was grown in SDC-expressing Dcp2-GFP and washed and grown in media without glucose or with 1 M KCl as indicated. The percentage of cells (>100) containing P bodies is indicated on the bottom left of each image using an automatic Otsu threshold. Scale bars = 3 μm. The intensity of the GFP signal is indicated below. (**B**) Left: Box plots showing the quantification of the P bodies in the wild-type and *edc3∆ lsm4∆C* strains (line, median; box 25^th^ and 75^th^ percentiles; whiskers, 10^th^ and 90^th^ percentiles, n = 50). The absolute number of P bodies was calculated from sum-projected Z stacks. Right: Quantification of the area-intensity of an average P body in the wild-type and mutant strains is in arbitrary units. Error = 5–95% confidence interval.

The *edc3∆ lsm4∆*C mutant has been reported previously as being defective in P body assembly under stress^19^,^22^. We found no difference in growth between the wild type and mutant strains both without stress and with osmotic stress (Fig. S1). We next investigated the two strains’ ability to form P bodies. First, we quantified cells for P bodies as defined by Dcp2-GFP foci under unstressed and stressed conditions. We examined cells under glucose deprivation and osmotic stress. The P body sizes in the wild-type cells and the *edc3∆ lsm4∆C* mutant were quantified for area, intensity and number per cell to provide a consistent assessment of P body levels^23^. Within fifteen minutes, the wild-type strain had P bodies in more than 78% of the cells under both glucose starvation and osmotic stress, whereas the *edc3∆ lsm4∆C* mutant did not (Fig. 1A,B). Even after 30 minutes, we observed only a very limited number of P bodies after glucose deprivation in the *edc3∆ lsm4∆C* mutant. These data support the notion previously put forward that the *edc3∆ lsm4∆C* strain is a *bona fide* P body mutant^19^,^22^,^24^.

### mRNA stabilization under osmotic stress is limited in the *edc3∆ lsm4∆C* mutant

**Figure 2:**
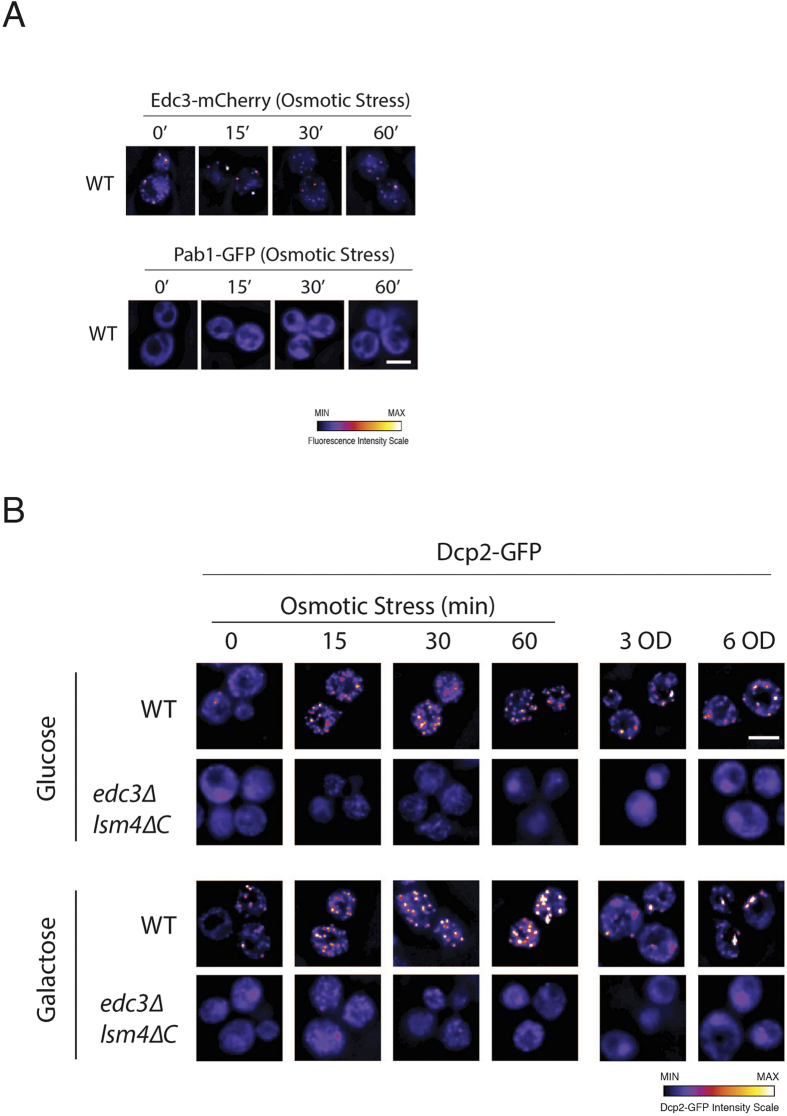
P bodies are induced by osmotic stress in the wild-type yeast but are greatly reduced or absent in the *edc3∆ lsm4∆C* mutant. (**A**) Wild-type cells with the pTN106 plasmid expressing Edc3-mCherry and Pab1-GFP. The cells were grown in SD −Ura + 2% glucose. The cells were washed and grown under osmotic stress in SD −Ura + 1 M KCl + 2% glucose for the indicated time. All images have the same contrast range. The scale bars represent 3 μm. The intensity of the GFP signal is indicated below. (**B**) Yeast cells expressing Dcp2-GFP at its endogenous locus bodies for wild-type and *edc3∆ lsm4∆C* mutant cells during the osmotic stress time course and high cell density. The cells were grown in SD media plus 2% galactose or 2% glucose as indicated. Osmotic stress was applied by the washing and addition of SDC + 1 M KCl + 2% glucose for the time indicated. All of the images have the same contrast range. The scale bars represent 3 μm. The intensity of the GFP signal is indicated below.

We next wished to examine whether Edc3 and the glutamine/asparagine rich C-terminus of Lsm4 would be important for the stabilization of mRNAs in response to stress. We chose to examine stabilization under osmotic stress for two reasons. First, high osmolarity is a key stress in yeast^2^. Second, osmotic stress induces P bodies but not stress granules^25^, which we confirmed in our experimental conditions (Fig. 2A,B). The *edc3∆ lsm4∆C* mutant is believed to reduce stress granules as a consequence of its inability to form P bodies^22^.

**Figure 3:**
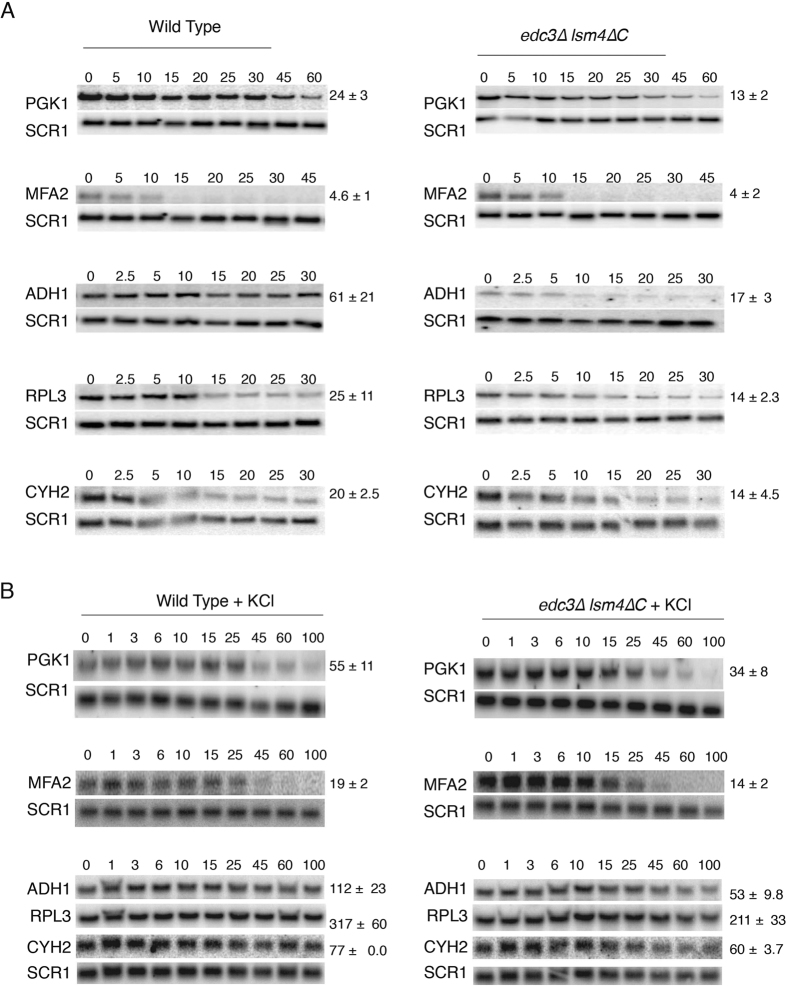
mRNA stability under unstressed and osmotic stressed conditions in the wild-type and *edc3∆ lsm4∆C* strains. (**A**) Northern blots for the half-life of the *PGK1* and *MFA2* mRNAs in the wild-type and *edc3∆ lsm4∆C* mutant cells. Time points after transcriptional shut-off by glucose addition are shown. *SCR1* is the loading control. Error = SD, *PGK1* n = 3, *MFA2* n = 3. The *ADH1* (n = 3), *RPL3* (n = 4) and *CYH2* (n = 3) mRNAs are depicted after thiolutin shut-off. Blots were cropped for figure construction. Error = SD. (**B**) As above after the addition of 1 M KCl to induce osmotic stress. *PGK1* n = 3, *MFA2* n = 4, *ADH1* n = 3, *CYH2/RPL28* n = 3, and *RPL3* n = 3.

We previously observed that mRNA half-lives were reduced in the *edc3∆ lsm4∆C* mutant compared to the wild-type yeast under unstressed conditions^21^. We confirmed these results by examining the *PGK1* and *MFA2* mRNA half-lives in both strains under the control of the *GAL1* UAS integrated into the *CUP1* locus^26^. We determined the mRNA half-lives after the induction of transcription by growing the yeast using galactose as a carbon source. Transcription was subsequently inhibited by addition of glucose. Cells were harvested at multiple time points after transcriptional inhibition, and the mRNA half-lives were determined (Fig. 3A). As we previously observed, both *PGK1* and *MFA2* had shorter half-lives in the *edc3∆ lsm4∆C* mutant (Fig. 3A). The reduction in mRNA half-life was significant for *PGK1*. We wished to confirm if other mRNAs are more stabilized in the wild-type than the *edc3∆ lsm4∆*C mutant strain. We therefore examined the half-lives of the following three additional mRNAs: (1) *ADH1* and (2) *RPL3* and (3) *CYH2/RPL28*. Again, we observed the same trend in the *edc3∆ lsm4∆C* mutant (Fig. 3A). Similar to *PGK1*, the half-life of the more stable *ADH1* mRNA was significantly reduced in the *edc3∆ lsm4∆C* mutant.

**Figure 4:**
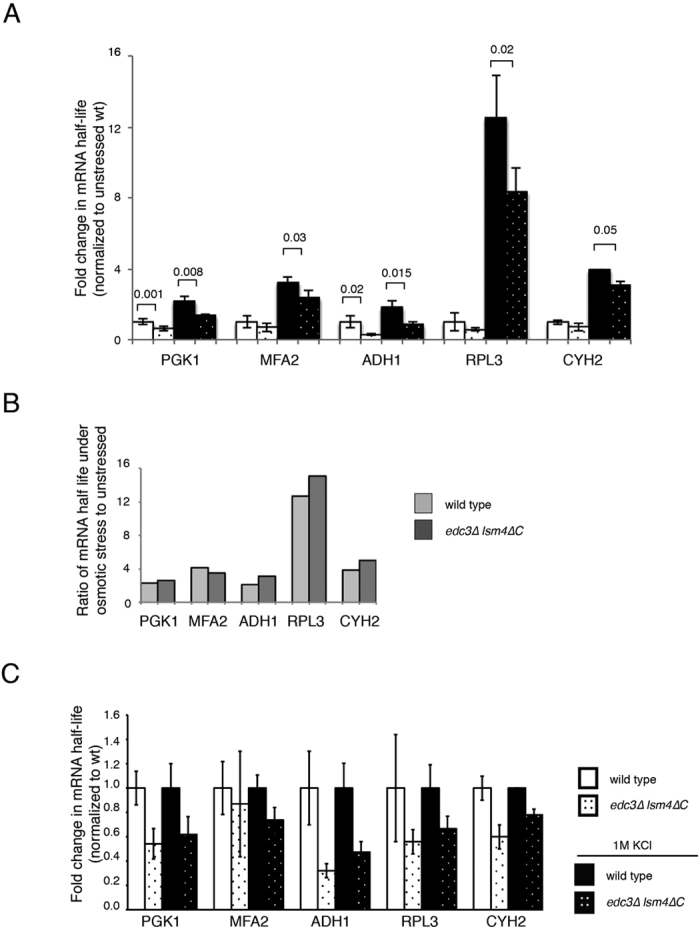
Fold stabilization of mRNA in the indicated strains normalized to unstressed background strain. (**A**) Bar graph for fold-mRNA stabilization under the conditions and using strains are indicated. Normalization is to the wild-type strain under unstressed conditions. Error = SD. Statistically significant pairings according to a t-test are indicated with their p values. (**B**) Data as above replotted to show the fold stabilization of the wild-type and *edc3∆ lsm4∆C* mutant under osmotic stress. (**C**) Data as above replotted normalized to the wild-type strain in either unstressed or osmotic stress (1 M KCl) conditions.

The rapid response to hyper-osmotic stress in yeast is a significant increase in mRNA stability^13^,^14^,^15^. We found that the half-lives for all five mRNAs tested were all significantly stabilized under hyper-osmotic stress with 1 M KCl (Figs 3B and 4A). Interestingly, the ribosomal protein-encoding mRNAs *CYH2/RPL28* and *RPL3* both were stabilized by an order of magnitude under osmotic stress in both the wild-type and mutant strains (Figs 3B and 4A). This is consistent with the observation that ribosomal protein-encoding mRNAs have unique behaviours under stress, where they are protected from decay early in the yeast stress response^7^,^27^. All these mRNAs had significantly reduced stability in the *edc3∆ lsm4∆C* mutant under hyper-osmotic stress compared to the wild-type yeast (Figs 3B and 4A). This includes mRNAs such as *MFA2, RPL3*, and *CYH2/RPL28*, which were not significantly destabilized without stress (Figs 3 and 4A). While the *edc3∆ lsm4∆C* limited the amount of stabilization, the fold stabilization when compared to the wild-type background was similar (Fig. 4B). These results demonstrate that the *edc3∆ lsm4∆C* mutant has reduced mRNA stability compared to the wild-type strain in both unstressed and hyper-osmotically stressed conditions (Fig. 4C).

### Poly(A) length is reduced in the *edc3∆ lsm4∆C* mutant under osmotic stress

**Figure 5:**
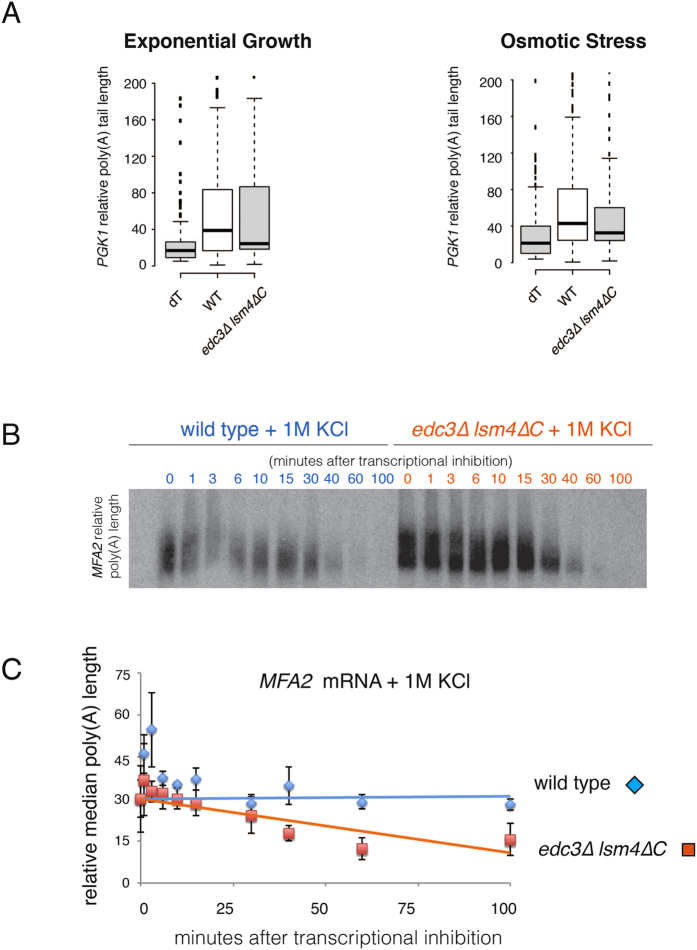
Roles of the exosome and deadenylase complex in the determination of mRNA stability. (**A**) Left: Poly(A) length of *PGK1* in yeast cells separated on a Urea/PAGE after RNase H digestion of the RNA from the indicated cell strains grown in YEP +2% galactose without additional stress. Quantification and binning was performed in Fiji. The oligo(dT) digestion control; wild-type mRNA; and *edc3∆ lsm4∆C* yeast strains are depicted. Centre lines = medians; box = 25^th^–75th percentiles as determined by R software; whiskers = 1.5 times the interquartile range from the 25^th^–75th percentiles, outliers = dots. n = 3. Right: RNA was extracted from wild-type and *edc3∆ lsm4∆C* cells grown in YEP +2% galactose and after 15 min growth with media supplemented with 1 M KCl. Isolated RNA was digested with RNase H and a *PGK1* specific primer (oTN273) was added to all reactions. An additional oligo(dT) was added to the reaction in the lane indicated. (**B**) Polyacrylamide gel and quantification of poly(A) length for *MFA2* mRNA transcriptional shut-off with concomitant osmotic stress (1 M KCl). The blot was cropped for figure construction. (**C**) The relative median poly(A) length plotted over time after transcriptional inhibition. The median was determined from the average of the medians histograms poly(A) intensity generated using Fiji plotted for the indicated time points. n = 2, error = SD.

To gain insight into whether deadenylation was affected in the *edc3∆ lsm4∆C* mutant, we examined the structure of the *PGK1* mRNA as it is significantly destabilized. Specifically, we determined the length of the poly(A) tail, the shortening of which is generally a prerequisite for subsequent degradation^17^,^28^. *PGK1* had a shorter poly(A) tail in the *edc3∆ lsm4∆C* mutant under both unstressed and hyper-osmotic conditions (Fig. 5A). This result is consistent with the shorter half-life for the *PGK1* mRNA, which can be attributed to more rapid deadenylation. We also examined the steady-state levels of the *MFA2* mRNA under exponential growth and osmotic stress and observed similar poly(A) tail lengths (data not shown). To more rigorously examine this mRNA, we performed a transcriptional shut-off experiment under osmotic stress (Fig. 5B). We next examined the median length of the poly(A) tails at the indicated times points after transcriptional inhibition (Fig. 5B). Our data show that the median poly(A) length of *MFA2* shortened more rapidly in the *edc3∆ lsm4∆C* mutant than in the wild-type strain, most dramatically after the 15 minute time-point (Fig. 5C). These data support a model whereby the reduced mRNA stability in the *edc3∆ lsm4∆C* mutant under hyper-osmotic stress is due to more rapid deadenylation.

### Limited stress-induced mRNA stabilization in the *edc3∆ lsm4∆C* mutant is consistent with increased deadenylation

**Figure 6:**
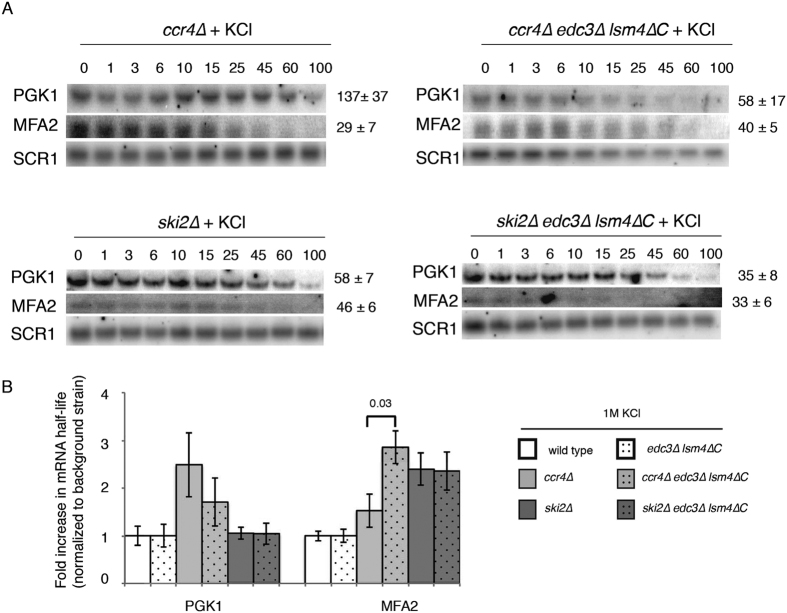
mRNA stability in *ccr4∆* and *ski2∆* backgrounds. (**A**) Northern blots for the half-life of the *PGK1* and *MFA2* mRNAs in the WT and *edc3∆ lsm4∆C* mutant cells in the *ccr4∆* and *ski2∆* backgrounds as indicated. Time points after transcriptional shut-off by glucose addition are shown. *SCR1* is the loading control. Blots were cropped for figure construction. Error = SD, *ccr4∆* background: *PGK1* n = 3, *MFA2* n = 3 (wt), 2 (*edc3∆ lsm4∆C*); ski2∆ background: *PGK1* n = 2. (**B**) Bar graph of *PGK1* and *MFA2* mRNA half-lives in the indicated strains. Half-life normalization is to the wild-type or *edc3∆ lsm4∆C* strains, respectively. Statistically significant pairings according to the t test are indicated with their p values. Error = SD.

We wished to get insight into in the relative importance of mRNA degradation pathways in absence of Edc3 and the glutamine/asparagine rich domain of Lsm4. To this end, we examined the half-life of the *MFA2* and *PGK1* mRNAs in the background of mutants defective in key mRNA degradation pathways in yeast. Previous experiments have shown that mRNAs that have greater dependence on a decay pathway show a concomitant greater increase in mRNA stability in the corresponding decay mutant^29^. We determined the mRNA stabilities in a deadenylase (*ccr4∆*) and an exosome (*ski2∆*) mutant. However, we did not use a mutant from the decapping-dependent pathway, *xrn1∆*, as it already displays exceptionally long half-lives for the mRNAs we are examining without osmotic stress^21^. Because both the *PGK1* and *MFA2* poly(A) tails were shorter at steady-state or had faster deadenylation, we examined whether the Ccr4/Not complex was acting to limit hyper-osmotic shock-induced mRNA stabilization. Ccr4 is the primary enzyme for cytoplasmic deadenylation in yeast and is required for catalytic activity of the Ccr4/Not complex^30^. In the absence of Ccr4 in the *edc3∆ lsm4∆C* strain when compared to *ccr4∆, MFA2* was significantly stabilized, whereas *PGK1* surprisingly exhibited the opposite effect (Fig. 6). Nevertheless, previous work on mRNA stability in osmotic stress attributed the normally minor Pan2/3 complex to significantly affect deadenylation^13^. When mRNA decapping-dependent decay is the only functional pathway after deadenylation, as in the *ski2∆* mutant, there was no significant difference between the wild-type and *edc3∆ lsm4∆C* strains, suggesting that both the dependence on the exosome as well as the decapping-dependent pathways is similar under osmotic stress in the two strains (Fig. 6).

### *In vivo* decapping assay suggests that the *edc3∆ lsm4∆C* mutant may differentially affect full-length mRNAs and mRNA degradation intermediates

**Figure 7:**
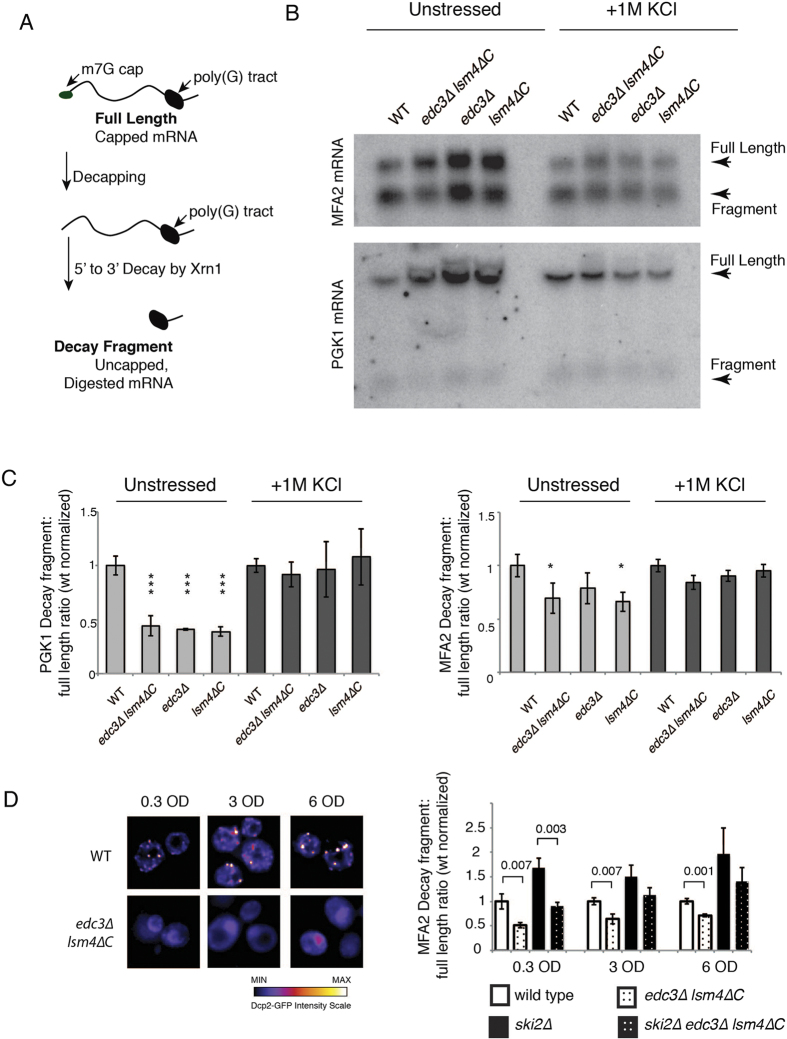
*In vivo* mRNA decapping assay for unstressed and stationary phase cells. (**A**) Depiction of the full-length capped mRNA and the decay fragment generated by decapping and 5′-to-3′ degradation, which is ultimately blocked by the poly(G) tract inserted into the 3′ UTR. (**B**) Northern blots depicting the expression of the *MFA2* and *PGK1* mRNAs expressed from the *GAL1* promoter in cells grown to midlog phase in YEP galactose. The upper band in each case is the full-length mRNA and the lower is the decapped mRNA decay fragment blocked by the poly(G) tract in the 3′ UTR. Blots were cropped for figure construction. (**C**) The relative fragment to full-length mRNA ratio was determined for *MFA2* and *PGK1* in the indicated strains. Error = SD, n = 3. Significance determined in comparison to wild-type by student’s t test. *p < 0.05, **p < 0.01, and ***p < 0.001. (**D**) Left: Wild-type and *edc3∆ lsm4∆C* strains were grown in SDC expressing Dcp2-GFP, harvested and imaged at 0.3, 3 and 6 OD. The intensity of the GFP signal is indicated below. Right: The relative fragment to full-length mRNA ratio was determined for *MFA2* and *PGK1* in the indicated strains. Statistically significant pairings according to a t test are indicated with their p values. Error = SD, n = 3.

We next wished to assess *in vivo* mRNA decapping in the wild-type and *edc3∆ lsm4∆C* mutants, including the individual *edc3∆* and *lsm4∆C* mutants. *In vivo* mRNA decapping rates can be determined by assessing the ratio of uncapped mRNA decay intermediates to full-length mRNAs^31^. The mRNA decay intermediate is generated after mRNA decapping and 5′-to-3′ exonuclease digestion by the cytoplasmic exonuclease Xrn1 (Fig. 7A). The 5′-to-3′ digestion of the mRNA by Xrn1 can be blocked by secondary structure. In these experiments, it is sterically blocked by a poly(G) tract in the 3′-UTR of the *MFA2* and *PGK1* mRNAs^26^. This results in the generation of a stable decay intermediate or fragment that can be further degraded from its 3′-end by the cytoplasmic exosome (Fig. S2)^29^,^32^. The degradation of the fragment by the 3′-to-5′ exonucleotic activity of the exosome results in the *PGK1* and *MFA2* decay fragments having half-lives of approximately 15 minutes^32^. This ratio has been used to assess mRNA decapping as the fragment to full-length mRNA ratio is proportional to mRNA decapping^31^.

We first examined the *PGK1* mRNA, which is degraded at roughly equal rates by both the 5′-to-3′ decapping-dependent and 3′-to-5′ exosome-mediated pathways^29^. Under unstressed conditions, all the decapping mutants we examined (*lsm4∆*C, *edc3∆*, and *edc3∆ lsm4∆*C) had a fragment to full length ratio suggestive of slower decapping (Fig. 7B,C and S2). Both Edc3 and Lsm4 are factors involved in enhancing mRNA degradation^33^,^34^. As such, mutants in these proteins would be expected to increase mRNA half-life. Reduced decapping in the single decapping mutants (*edc3∆* and *lsm4∆C*) is consistent with the longer half-life observed in the mRNAs examined for the *lsm4∆C* and *edc3∆* strains^19^,^21^,^35^. However, the *edc3∆ lsm4∆C* mutant has a significantly reduced half-life (Fig. 3A), which is not consistent with reduced mRNA decapping. In contrast, when we examined the decay fragment to full length mRNA ratio under osmotic stress, we observed no differences in apparent *in vivo* decapping (Fig. 7C). We attribute this result to the slower deadenylation observed under osmotic stress (Fig. 5), which is normally a pre-requisite for mRNA decapping^36^.

We next examined the *MFA2* mRNA under unstressed conditions, which is primarily degraded by the decapping-dependent pathway^29^. We found that both the *edc3∆ lsm4∆C* and *lsm4∆C* mutants had a significantly lower relative amount of fragment, while *edc3∆* was not significantly lower. Our fragment to full-length data are consistent with our previous determination of mRNA stability in the *edc3∆* and the *lsm4∆C* mutants^21^. The *edc3∆* mutant had no change in decapping ratio nor in mRNA half-life while the *lsm4∆C* mutant had decreased mRNA decapping and increased mRNA half-life. In contrast, the *edc3∆ lsm4∆C* double mutant had an apparent slower decapping combined with a shorter half-life (Figs 7C and 3A), as seen for the *PGK1* mRNA.

These results taken together suggest that under unstressed conditions the single mRNA decapping mutants’ apparent *in vivo* decapping rate reflects the observed mRNA half-life. In contrast, the *edc3∆ lsm4∆C* strain has slower apparent *in vivo* mRNA decapping coupled to faster mRNA degradation, which could be due to the action of other mRNA decay pathways (see next section). Finally, under osmotic stress the apparent *in vivo* mRNA decapping is similar in all strains (Fig. 7C) consistent with a reduction in deadenylation as it is generally a pre-requisite for both 5′-to-3′ decapping-dependent and 3′-to-5′ exosome-mediated degradation (Fig. 5C).

### The cytoplasmic exosome can affect the abundance of mRNA decay intermediates

The *in vivo* mRNA decapping assay (fragment to full-length mRNA ratio) revealed that the *edc3∆ lsm4∆C* strain displays slower apparent decapping (Fig. 7C), while having faster mRNA degradation (Fig. 3A). One possibility that may reconcile these results is that the *edc3∆ lsm4∆C* uses another mRNA degradation pathway to reduce the abundance of the RNA decay fragment. A potential possibility is the exosome, which is excluded from P bodies^37^,^38^. While the primary mechanism of mRNA degradation in yeast is 5′-to-3′, deadenylated mRNA can also be degraded from the 3′ end by the exosome (Fig. 7A). If the *edc3∆ lsm4∆C* mutant results in more active exosome activity on the decay fragment, then its abundance would be reduced. This would appear as slower decapping, but could result from a greater contribution of exosome-mediated degradation.

To examine this possibility, we first determined the effect on apparent *in vivo* mRNA decapping (mRNA decay fragment to full length ratio) when the exosome activity is removed. We used the *ski2∆* mutant, a component of the Ski2/3/8 complex, which is defective in cytoplasmic exosome-mediated degradation^32^. The decay fragment is significantly more stable and abundant in the *ski2∆* mutant^32^. We examined this effect with the *MFA2* mRNA as the degradation of the decay intermediate by the exosome has been calculated to be an order of magnitude slower than the decapping and 5′-to-3′ degradation that generated it^29^.

By examining the fragment to full-length *MFA2* mRNA ratio for *ski2∆* mutants in the wild-type and *edc3∆ lsm4∆C* backgrounds, we found two major results (Fig. 7D). First, the *edc3∆ lsm4∆C* mutant displayed a significantly lower relative amount of fragment compared to the wild-type yeast as we observed previously (Fig. 7C,D). Second, there was still a significantly lower ratio of decay fragment in the background of the *ski2∆* mutant deficient in exosome-mediated degradation. This suggests that while the exosome contributes to degradation of the fragment, the *edc3∆ lsm4∆C* mutant could have residual 3′-to-5′ degradation still occurring. Consistent with this possibility, 3′-to-5′ nibbling and degradation of unknown origin has been observed in the individual *ski* mutants as well as in the combined *ski2∆ ski3∆ ski7∆ ski8∆* mutants^39^.

Messenger RNA decay intermediates are more localized to P bodies than full length mRNA^38^. Furthermore, failure to degrade in the 5′-to-3′ direction due to RNA structure results in decay fragment accumulation in P bodies, most significantly when overexpressed^27^,^40^,^41^. We therefore next considered a condition in which P bodies are more prominent, such as during the diauxic shift at higher ODs (Fig. 7D). At an OD of 3 and 6, we again found that the *edc3∆ lsm4∆C* mutant had a significantly lower ratio of fragment to the full-length *MFA2* mRNA as compared to the wild-type yeast. In contrast to exponential growth, however, the fragment to full-length *MFA2* mRNA levels were not significantly different between the wild-type and the *edc3∆ lsm4∆C* mutant in the absence of the Ski2 protein during diauxic shift (Fig. 7D). While these results are suggestive of an increased role of the exosome in fragment degradation with larger P bodies, the absence of significance differences could be due to alternative effects, which are discussed below.

## Discussion

Our findings in this study demonstrate that the *edc3∆ lsm4∆C* strain defective in P body formation displays reduced mRNA stabilization in response to osmotic stress. We previously demonstrated that this mutant can destabilize some mRNAs while not affecting others without stress. Under osmotic stress from 1 M KCl, every mRNA we examined in the *edc3∆ lsm4∆C* mutant showed reduced stability (Fig. 4A). We provide evidence that the poly(A) length of the *MFA2* and *PGK1* mRNAs are reduced in the *edc3∆ lsm4∆C* mutant upon osmotic stress (Fig. 5). Since deadenylation is generally the first and rate limiting step of mRNA degradation in yeast, more rapid shortening of the poly(A) tails alone can result in faster degradation^42^.

The Ccr4/Not complex is the major cytoplasmic deadenylase in yeast^42^. More rapid deadenylation by the Ccr4/Not complex in the *edc3∆ lsm4∆*C mutant is supported by the observation that the *MFA2* mRNA is significantly stabilized in the *ccr4∆ edc3∆ lsm4∆C* strain compared to the *ccr4∆* single mutant (Fig. 6). We observed a similar effect for the *PGK1* mRNA in the *ccr4∆ edc3∆ lsm4∆C* mutant under unstressed conditions^21^. Interestingly, the *PGK1* mRNA did not exhibit a similar defect under osmotic stress (Fig. 6). Because the steady-state poly(A) length is shorter for the *PGK1* mRNA, the result could be due to another deadenylase, such as Pan2/3 complex, facilitating the more rapid deadenylation in the *edc3∆ lsm4∆C* mutant under osmotic stress. Consistent with such a model, Pan2/3 has been reported to play a more significant role in deadenylation during osmotic stress^13^.

How might deadenylation be affected in the *edc3∆ lsm4∆C* mutant? We can envision three scenarios. First, the abundance of mRNA degradation factors could be altered. For example, we found that the Ccr4 protein is significantly more abundant in the *edc3∆ lsm4∆C* mutant when grown in galactose as a carbon source^21^. In contrast, when grown in glucose, the abundance of the Ccr4 protein is similar in the wild-type and *edc3∆ lsm4∆C* strains^21^. Since the mRNA half-lives when grown in both carbon sources exhibited a reduction in osmotic stress-induced stabilization in the *edc3∆ lsm4∆C* mutant, these data suggest that it may not be due to increased abundance of the Ccr4 protein (Fig. 3B). Second, the assembly of mRNPs that promote mRNA degradation may affect mRNA stability under osmotic stress. The *edc3∆ lsm4∆*C strain is mutated in two decapping-activating proteins. *A priori*, one would expect that defective mRNA decapping would promote mRNA stability instead of the destabilization that we observe (Fig. 3). One possibility is suggested by the recent links between the deadenylation complex and the decapping-activating protein Pat1^43^,^44^,^45^,^46^. It is possible that the absence of Edc3 and the Q/N-rich Lsm4 C-terminus affects the assembly of a deadenylation complex on mRNPs, perhaps allowing the early precocious assembly of deadenylation complexes on the mRNA. A third alternative possibility for the limitation of osmotic stress-induced mRNA stabilization in the *edc3∆ lsm4∆C* mutant is due to the reduced formation of P bodies in this strain. Both this work (Fig. 1) and that of others have shown that P bodies are eliminated or greatly reduced upon osmotic stress in the *edc3∆ lsm4∆C* mutant^19^,^22^. When P bodies are formed, the degrading mRNAs and deadenylation complexes may be spatially separated. For example, both the Ccr4/Not and Pan2/3 deadenylases have been found to be localized within P bodies^16^,^17^,^18^,^20^. Recent studies have also demonstrated that a significant portion of mRNA can undergo degradation during translation^47^,^48^. If there is a reduced cytosolic concentration of deadenylases due to sequestration within P bodies, the cytosolic mRNA may be subjected to reduced mRNA degradation rates. Similarly, in the *hog1∆* mutant, P bodies are more intense when exposed to osmotic stress and mRNA becomes stabilized to a greater extent than in wild-type yeast^14^.

Alternatively, it is possible that the accumulation of deadenylated mRNA that we observed is a consequence of reduced decapping^31^,^34^,^49^. However, given that the mRNA half-lives are shorter in the *edc3∆ lsm4∆C* mutant, more rapidly shortened poly(A) tails are more consistent with faster deadenylation. This model is supported by the mRNA stability of both the wild-type yeast and the *edc3∆ lsm4∆C* mutant combined with the exosome mutant *ski2∆* (Fig. 6). The stability is not significantly altered between the wild-type and *edc3∆ lsm4∆C* strains suggesting that the relative contribution of decapping-dependent degradation is similar in both strains (Fig. 6). Specifically, the relative stabilization of the *ski2* mutant in both strains is suggestive of similar 3′-to-5′ exosome contributions and thus similar contributions from the decapping-dependent pathway^29^.

Our data also provides support for alteration of the mechanism of degradation of mRNA found in P bodies. A significant source of P body bound mRNA can be generated by the addition of secondary structure blocking further 5′-to-3′ degradation^27^,^40^,^41^,^50^. In our experiments, the *MFA2* and *PGK1* mRNAs have poly(G) tracts in their 3′UTR (Fig. 7), which result in accumulation of decay intermediates^27^,^51^. These decay intermediates accumulate in P bodies, while the 3′-to-5′ exonuclease complex (the exosome) is excluded from P bodies^52^. It is therefore plausible that P bodies can restrict access of the exosome to the mRNAs found in P bodies. Consistent with such a possibility, we find that the abundance of decay intermediates is reduced in the *edc3∆ lsm4∆C* mutant unable to form P bodies (Fig. 7B). When the exosome is inactivated, such as in the *ski2∆* mutant, we find an increase in the amount of decay fragment in the wild-type strain and a reduced increase in the *edc3∆ lsm4∆C* mutant, which are significantly different (Fig. 7D). In contrast, we found no significant differences during the diauxic shift, where P bodies are more prominent. This may indicate that the model of P bodies providing protection from the exosome is incorrect. However, there can be at least three alternative effects which could account for this result. First, exonucleolytic 3′-to-5′ nibbling could be occurring independently of the Ski complex^39^. Second, we also observe no fragment increase between the wild-type and *edc3∆ lsm4∆C* mutant in response to hyper-osmotic stress (Fig. 7C). This effect is likely correlated with the inhibition of deadenylation^13^. Such an effect may be occurring by altering deadenylation or the activity of the exosome, although the *PGK1* and *MFA2* mRNAs were determined to have the same half-lives during exponential growth and in stationary phase^53^. A third possibly is that after diauxic shift, the P body bound mRNAs are part of a pool of mRNAs that can only be extracted with proteases^54^. Such a pool would not be represented in our experiments. Therefore, future experiments will be necessary to examine how P bodies effect the mRNA that are bound within these RNA granules.

**Figure 8:**
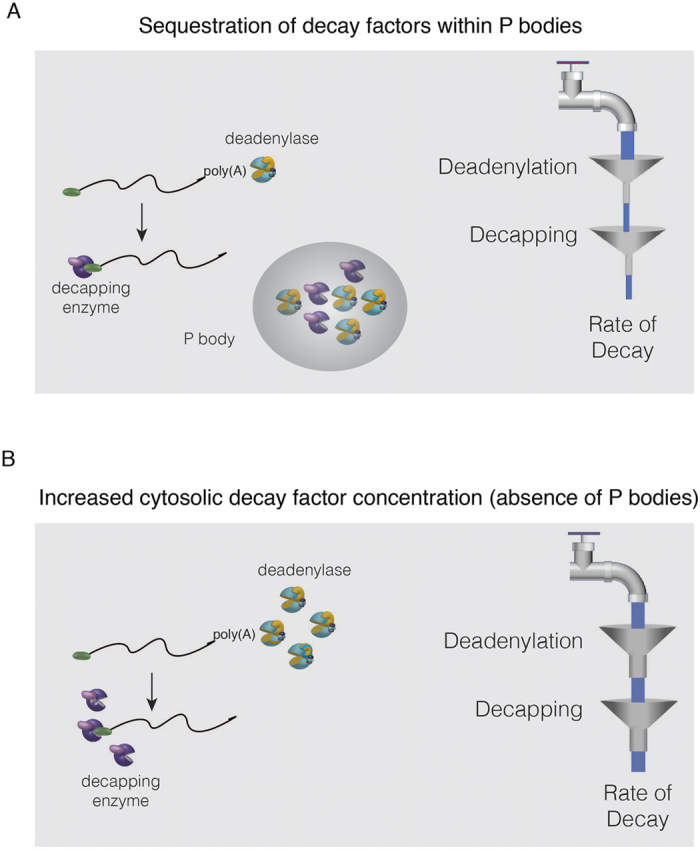
Model for a mechanism by which P bodies can influence mRNA degradation under stress. (**A**) Left: When P bodies can form in response to stress, mRNA decay factors accumulate in P bodies reducing their concentration in the cytosol. Right: Model of the effect of P bodies on degradation. The amount of mRNA subject to degradation in yeast has to generally undergo two sequential steps: deadenylation and decapping. With the reduction in the cytosolic concentration of deadenylase complexes, there is a concomitant reduction in deadenylation, resulting in a reduced net rate of mRNA degradation. (**B**) When P bodies are absent the rate of degradation is increased due to faster deadenylation from the higher cytosolic concentration due to absence of sequestration within P bodies.

In conclusion, we favour a model supported by our data in which mRNAs are generally subjected to more rapid deadenylation in the *edc3∆ lsm4∆C* mutant. As a consequence, mRNAs are less stable due to the greater flux of deadenylated mRNA delivered to the decapping enzyme. This model can be depicted as a stream of water from a faucet representing the pool of cytoplasmic mRNA (Fig. 8). This pool of mRNA is subjected to two enzymatic bottlenecks: deadenylation and decapping (represented by funnels in Fig. 8). When P bodies are induced by osmotic stress, deadenylation and decapping complexes accumulate within the RNA granules, reducing their cytosolic concentration. This has the effect of slowing the bottlenecks of deadenylation and decapping (represented by small funnel diameters) and reducing the net rate of mRNA degradation (Fig. 8A). Inability to form P bodies in response to osmotic stress results in a higher cytosolic concentration of mRNA decay factors (Fig. 8B). This increases the flux possible to subsequent 5′-to-3′ degradation after decapping and thus reduced mRNA stability.

## Materials and Methods

### Yeast strains and growth conditions

The genotypes of all of the strains used in this study are listed in Table S1. Strains were grown using either yeast extract/peptone (YP) medium or synthetic medium complete media (SDC) lacking amino acids as indicated. The media contained 2% galactose as a carbon source for glucose transcriptional shut-off experiments; otherwise, 2% glucose was used. The strains were grown at 30 °C. Where indicated, transcription was halted by the addition of 6 μg ml^−1^ thiolutin. The thiolutin concentration was titrated to be the minimal level to inhibit transcription^55^. For glucose depletion and osmotic stress studies, exponentially grown yeast were centrifuged, washed in the appropriate media (either lacking glucose or with the addition of 1 M KCl) and resuspended in 10 ml of stress-inducing media. The cells were harvested after the indicated period of growth with aeration.

Yeast genomic knock-out strains were generated using homologous recombination with regions of homology approximately 50 nucleotides upstream of the ATG and 50 nucleotides downstream of the stop codon. The gene of interest was replaced with the nourseothricin (*natNT2*) and hygromycin B (*hphMX4*) antibiotic resistance genes as described previously^56^. The sequences present in the knockout strains were confirmed by PCR.

### Microscopy

Live yeast cells were resuspended in appropriate minimal media and visualised using a Deltavision Spectric microscope (GE Healthcare) with an Olympus 60 × 1.4NA objective without binning. Microscopic images were deconvolved using the classical maximum likelihood estimation algorithm in Huygens Essential 4.4 (SVI, Hilversum, Netherlands), except those deconvolved using the Deltavision standard settings as noted in the figure legends. Each of the resulting images was depicted by means of sum intensity projections from a Z series of 20 0.25-μm thickness slices displayed with Fiji^57^. The images in each panel are within the same contrast range displayed using the Fire lookup table in Fiji. The quantification of the number, size and intensity-area of the P bodies was performed with the Fiji software package^57^ as described previously^23^. This results in an unbiased analysis of the P bodies; however, the values obtained are usually lower than those that can be detected by visual examination^58^. Briefly, Z-stacks were sum projected, and the background was subtracted and smoothed. The resulting images were processed automatically using Otsu thresholding^59^. The individual foci were counted in approximately 50 cells for each condition and strain, taking into account only foci between 7 and 500 pixels in area. The intensities of the foci were quantified with reference to the non-threshold sum projection. The area-intensity of each P body was determined by multiplying the mean area of the P body by its mean fluorescence intensity. The co-localization of proteins was with the plasmids detailed in the figure legends and Table S3.

### RNA Analysis

RNA was purified by lysing yeast cells with glass beads; this was followed by a phenol/chloroform/isoamyl alcohol extraction and ethanol precipitation. Northern blots were probed with the oligonucleotides listed in Table S2. The mRNA half-lives were determined using transcriptional shut-off with 6 μg ml^−1^ thiolutin or by the use of an inducible *GAL* promoter integrated into the genome, which controls the *MFA2*pG and *PGK1*pG mRNAs. The optimal thiolutin concentration was obtained by titration and half-life determination^55^. Transcription from the *GAL* promoter was repressed by the addition of 4% dextrose to the cell medium after washing as indicated. Yeast were grown in SDC-Trp for the unstressed half-life determinations for Fig. 3A using thiolutin shut-off (i.e. *ADH1, RPL3* and *CYH2*), otherwise in YEP with the indicated carbon source. The quantification of the northern blot bands was performed with Quantity One (Bio-Rad). The mRNA half-lives were determined by the best linear fits of the mRNA band intensities normalized to the *SCR1* loading control. The mRNA half-lives were subjected to two-tailed unpaired Student’s t tests. Statistical significance was determined with a p-value cut-off of 0.05. The oligonucleotides used in this study are indicated in Table S2. RNase H reactions were performed as described using oligo(dT)18 (ThermoFisher) and/or oTN273 as indicated^60^.

## Additional Information

**How to cite this article:** Huch, S. and Nissan, T. An mRNA decapping mutant deficient in P body assembly limits mRNA stabilization in response to osmotic stress. *Sci. Rep.* **7**, 44395; doi: 10.1038/srep44395 (2017).

**Publisher's note:** Springer Nature remains neutral with regard to jurisdictional claims in published maps and institutional affiliations.

## Supplementary Material

Supplementary Information
