# Supplementary material for: An mRNA decapping mutant deficient in P body assembly limits mRNA stabilization in response to osmotic stress
Source: Sci Rep. 2017 Mar 14;7:44395. doi: 10.1038/srep44395 (PMC5349606; doi:10.1038/srep44395)
Supplement: Supplementary Information [file srep44395-s1.pdf]

An mRNA decapping mutant deficient in P body assembly limits mRNA stabilization in response to osmotic stress

SUPPLEMENTAL INFORMATION

Susanne Huch and Tracy Nissan

Supplementary Information

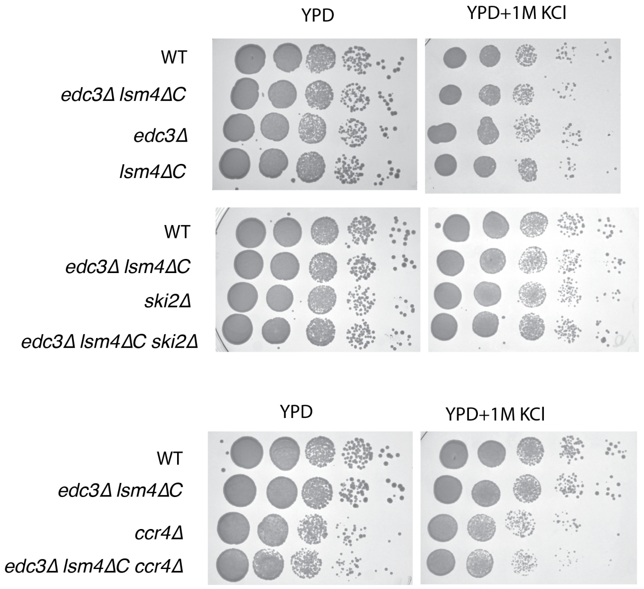

Figure S1. **Characterization of growth of the *edc3*Δ *lsm4*Δ*C* mutant.** Growth assay for wild-type yeast, the *edc3*Δ *lsm4*Δ*C* and individual mutation strains on YPD or YPD supplemented with 1 M KCl. Strains were grown for three or four days for YPD and YPD + 1M KCl respectively.

A

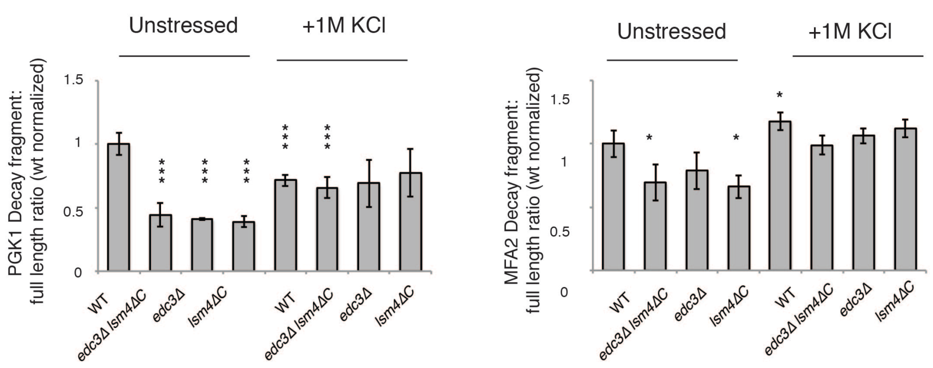

B

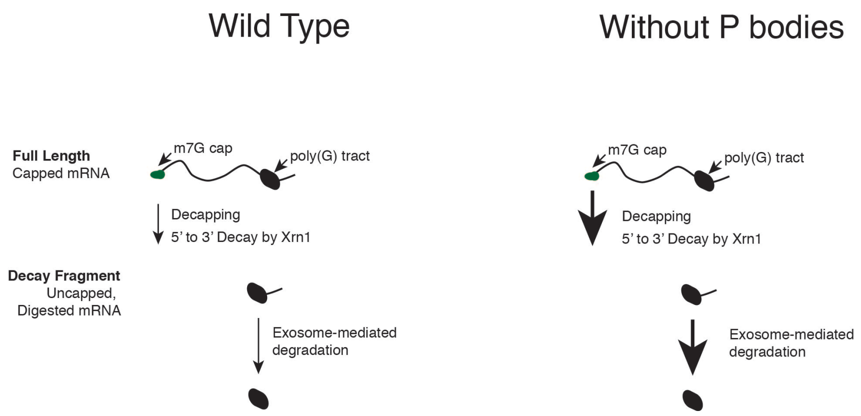

Figure S2. Fold stabilization of mRNA in the indicated strains normalized to unstressed background strain and model for exosome-mediated degradation. (A) Bar graph for fold-mRNA stabilization under the conditions and using strains are indicated. Normalization is to the wild-type strain under unstressed or stressed conditions as indicated. Error = SD. Statistically significant pairings according to a t-test are indicated with their p values. **(B)** Depiction of the full-length capped mRNA and the decay fragment generated by decapping and 5'-to-3' degradation, which can be further degraded by the exosome. Since the fragment is not sequestered in the exosome, it is degraded faster in the strain without P bodies (indicated by the larger arrows).

Table S1. **Strains used in this study.**

Strain Number	Genotype	Reference
yTN29	*MATalpha leu- cup1::LEU2/PGK1pG/MFA2pG lys2-201 trp- ura-*	1
yTN38	*MATa ura3-52 leu2-2, 112 trp1-Δ1 cup1::LEU2/PGK1pG/MFA2pG dcp1::URA3 lys2*	2
yTN45	*MATa his4 leu2 lys2 trp1 ura3 cup1::LEU2/PGK1pG/MFA2pG dcp2::TRP1*	2
yTN86	*MATalpha leu2 trp1 ura3 his4 cup1::LEU2/PGK1pG/MFA2pG lsm4Δc:: kanMX6 edc3::kanMX6*	2
yTN87	*MATalpha leu2 trp1 ura3 his4 cup1::LEU2/PGK1pG/MFA2pG lsm4Δc:: kanMX6 edc3::kanMX6 DCP2GFP:: kanMX6*	2
yTN150	*MATa leu- cup1::LEU2/PGK1pG/MFA2pG trp- ura- DCP2GFP:: kanMX6*	2
yTN177	*MATalpha leu2 trp1 ura3 lys2 cup1::LEU2/PGK1pG/MFA2pG ski2:: hphMX4*	3
yTN186	*MATalpha leu2 trp1 ura3 his4 cup1::LEU2/PGK1pG/MFA2pG lsm4Δc:: kanMX6 edc3::kanMX6 ski2:: hphMX4*	3
yTN190	*MATalpha leu2 trp1 ura3 his4 cup1::LEU2/PGK1pG/MFA2pG lsm4Δc:: kanMX6 edc3::kanMX6 ccr4:: hphMX4*	3
yTN246	*MATalpha leu2 trp1 ura3 lys2 cup1::LEU2/PGK1pG/MFA2pG edc3::NatMX*	3
yTN281	*MATalpha leu2 trp1 ura3 lys2 cup1::LEU2/PGK1pG/MFA2pG ccr4:: NatMX*	3
yTN378	*MATalpha leu2 trp1 ura3 lys2 cup1::LEU2/PGK1pG/MFA2pG lsm4Δc9xMyc::kanMX6*	3

Table S2. **Oligonucleotides used in this study.**

Oligo Number	**Sequence**	**Target Gene**
oTN100	GTCTAGCCGCGAGGAAGG	SCR1
oTN140	ATATTGATTAGATCAGGAATTCC	MFA2pG
oTN141	AATTGATCTATCGAGGAATTCC	PGK1pG
oTN270	GGCTGTAATGGCTTTCTGGTGGGATGGG	SCR1
oTN271	GGTTCAGGACACACTCCATCCCCG	SCR1
oTN273	AAGAAAGCAACACCTGGCAA	PGK1
oTN339	CCCATCTGTGGGTAACACCTTCGAAACCG	RPL3
oTN340	CCGGCCATACCTCTACCACCGGGG	CYH2
oTN350	CCAAGACTCTGTAACCCATAGCCTTGGC	ADH1
oTN489	CAATGGCCAGTCACCGTGCC	ADH1
oTN490	CACGCACACTACTCTCTAATGAG	ADH1
oTN491	CTGACAATGGTGGTCATACCAGCC	RPL3
oTN492	GAAGCGACGCACAACTGTTTTCC	RPL3
oTN707	ATTCAACAACACCACCAGCA	CYH2
oTN708	AGGATAGGAACGTACGTTATTATGCTTAATTAGTTCGATGGACC	CYH2

Table S3. **Plasmids used in this study.**

Plasmid Number	**Sequence**	**Reference**
**pTN106**	pPab1-GFP/Edc3-mCherry	[4]
pTN115	pRS315 TRP	[5]
pTN208	pEdc3-RFP	[6]
pTN196	pDcp2-RFP (URA3)	This work

Supplementary References

1. Hatfield, L., Beelman, C. A., Stevens, A. & Parker, R. Mutations in trans-acting factors affecting mRNA decapping in Saccharomyces cerevisiae. *Mol Cell Biol* **16,** 5830–5838 (1996).
2. Decker, C. J., Teixeira, D. & Parker, R. Edc3p and a glutamine/asparagine-rich domain of Lsm4p function in processing body assembly in Saccharomyces cerevisiae. *J Cell Biol* **179,** 437–449 (2007).
3. Huch, S. *et al.* The decapping activator Edc3 and the Q/N-rich domain of Lsm4 function together to enhance mRNA stability and alter mRNA decay pathway dependence in Saccharomyces cerevisiae. *Biol Open* **5,** 1388–1399 (2016).
4. Buchan, J. R., Muhlrad, D. & Parker, R. P bodies promote stress granule assembly in Saccharomyces cerevisiae. *J Cell Biol* **183,** 441–455 (2008).
5. Sikorski, R. S. & Hieter, P. A system of shuttle vectors and yeast host strains designed for efficient manipulation of DNA in Saccharomyces cerevisiae. *Genetics* **122,** 19–27 (1989).
6. Beckham, C. J. *et al.* Interactions between brome mosaic virus RNAs and cytoplasmic processing bodies. *J. Virol.* **81,** 9759–9768 (2007).
